# Primary extra-gastrointestinal stromal tumor of retroperitoneum: Clinicopathologic characteristics and prognosis of six cases

**DOI:** 10.3389/fonc.2023.1033598

**Published:** 2023-02-21

**Authors:** Jiaxin Lin, Weilin Liao, Jiahao Wang, Wenjuan Li, Xin Tang, Hongming Li, Xiaojiang Yi, Xinquan Lu, Zhaoyu Chen, Bosen Zhu, Xiaochuang Feng, Dechang Diao

**Affiliations:** ^1^ The Second School of Clinical Medicine, Guangzhou University of Chinese Medicine, Guangzhou, China; ^2^ Department of Colorectal (Tumor) Surgery, Guangdong Provincial Hospital of Chinese Medicine, Guangzhou, China

**Keywords:** extra-gastrointestinal stromal tumors, PDGFRA, KIT, retroperitoneal, sarcoma

## Abstract

**Aim:**

This study investigates the clinicopathological features and prognostic genic biomarker factors of primary retroperitoneal extra-gastrointestinal stromal tumors (EGISTs).

**Methods:**

The clinicopathological data of six patients with primary retroperitoneal EGIST were analyzed, including cell type (epithelioid or spindle), mitoses, and the presence of intratumoral necrosis and hemorrhage. Mitoses were counted and summed from 50 high power fields (HPFs). Mutations of exons 9, 10, 11, 13, 14, and 17 of the C-kit genes and those of exons 12 and 18 of the PDGFRA gene were examined. Follow-up was performed *via* telephone, and all outpatient records were reviewed. The last follow-up date was February 2022, the median follow-up was 27.5m and the postoperative status, medication, and survival of the patients were recorded.

**Result:**

The patients were treated with radical intent. Four cases (patients 3, 4, 5, and 6) underwent multivisceral resection for encroachment on the adjacent viscera. The postoperative pathological results demonstrated that all biopsy specimens were negative for S-100 and desmin, and positive for DOG1 and CD117. Additionally, four patients (case 1, 2, 4, and 5) were positive for CD34, four (case 1, 3, 5, and 6) were positive for SMA, four (case 1, 4, 5, and 6) had >5/50 HPFs, and three (case 1, 4, and 5) had Ki67 >5%. According to the modified National Institutes of Health (NIH) guidelines, all patients were graded as high-risk cases. By exome sequencing, exon11 mutations were detected in the six patients, while exon10 mutations were detected in two cases (patients 4 and 5). The median follow-up time was 30.5 (11–109) months, with only one fatality at 11 months.

**Conclusion:**

Retroperitoneal EGIST is a rare mesenchymal tumor that is difficult to distinguish from other retroperitoneal tumors. To diagnose this highly malignant tumor, low-threshold suspicion is necessary, and Kit and PDGFRA gene mutations should be routinely tested to confirm the diagnosis and guide subsequent treatment.

## Introduction

Extra-gastrointestinal stromal tumors (EGISTs) share the histopathological and immunohistochemistry characteristics of gastrointestinal stromal tumors (GISTs), predominantly within the peritoneum or retroperitoneum. The incidence of EGIST is approximately 5% of that of GIST, while retroperitoneal EGIST accounts for approximately 25% of that of EGIST (1). Because retroperitoneal EGIST is very rare, there is no unified understanding of its origin and mechanism. Retroperitoneal EGIST was first classified as leiomyosarcoma. It was not until 2001 that immunohistochemistry and electron microscopy provided evidence for the myogenic characteristics and neural properties of mesenchymal tumors, allowing the two to be distinguished. Although retroperitoneal EGIST has similar clinical features and different treatment strategies and prognoses to other retroperitoneal sarcomas, distinguishing between these tumors can be challenging (2–4).

EGISTs have certain tissue *immune markers*, including C-kit (CD117), CD34, and DOG-1; however, these are not specific, obstructing the differentiation of retroperitoneal EGISTs from other retroperitoneal sarcomas (5, 6). CD34 is only expressed in 60%–70% of GISTs, while other tumors, such as smooth muscle tumors, are immunopositive for CD34 (7). A focal positive for CD117 is also seen in some retroperitoneal leiomyosarcoma and liposarcoma tumor cells (8). In contrast, C-kit gene detection is a more efficient, sensitive, and reliable method for diagnosing retroperitoneal tumors. However, depending on the type of gene mutation, the pathophysiological hallmarks and clinical manifestations are different.

Additionally, there is a lack of consensus on treating this disease, owing to the rarity of cases. Surgery of retroperitoneal EGIST frequently refer to the treatment options of GIST. Localized GISTs are curable, with surgery as the standard treatment. Patients with GIST with KIT or PDGFRA mutations and sensitive to the tyrosine kinase inhibitor (TKI) at high risk of relapse have improved survival with adjuvant imatinib treatment. KIT-mutant EGISTs and GISTs, as well as some PDGFRA-mutant tumors, may respond to imatinib. However, the management of GIST, and decision on the neoadjuvant and adjuvant treatment of localized GIST at high risk of relapse, is based on the mutation analysis (9). Because most retroperitoneal sarcoma types are particularly insensitive to TKI, misdiagnosis results in the incorrect administration of treatments that can adversely affect the *prognosis* of patients. To better comprehend the pathogenesis and treatments of various diseases, it is necessary to determine the tumor characteristics. In this study, we describe the tumor characteristics of six cases of retroperitoneal EGIST and review the available literature.

## Methods

### Baseline characteristics

This retrospective analysis reviewed six consecutive patients with pathologically confirmed retroperitoneal EGIST treated at the Department of Gastrointestinal Tumor Center in Guangdong Provincial Hospital of Chinese Medicine between January 2011 and August 2018. Patient inclusion criteria were as follows: 1) stromal tumors diagnosed by histology and immunohistochemistry; 2) the main body of the tumor located in the retroperitoneum without gastrointestinal tract involvement, and 3) complete clinical data and follow-up data. The exclusion criteria were as follows: 1) patients with a history of GIST, and 2) pre-existing physical or mental disability or severe co-morbidity that may interfere with the outcome assessment. This study was reviewed and approved by the ethics committee of Guangdong Provincial Hospital of Chinese Medicine (ZE2022-025-01).

### Immunohistochemistry (IHC)

Immunohistochemistry (IHC) was performed according to manufacturers instructions. The sections were deparaffinized using xylene baths, antigens were retrieved in 10 mM citrate buffer then were blocked in 3% hydrogen peroxide for 15 min at 37°C. After incubating with blocking serum solution for 30 min at 37°C, these sections were incubated at 4°C through primary rabbit anti-human antibodies (CD34, CD117, DOG1, Ki67,S-100, SMA) Proteintech overnight, and incubated at secondary antibodies for 2 hours. Finally, the nuclei were counterstained with hematoxylin. Specimens were independently scored by two experienced pathologists. To assess the protein expression, the percentage of positive cells was calculated in five independent fields in higher-magnification objectives (× 400) that more than 50 cells. The final IHC score was a result of the positive cell ratio score.

### Sanger sequencing

PCR amplification products were purified using the PCR purification kit (Takara Japan). PCR amplification was performed for 30 cycles of pre-denaturation for 3 min at 98°C, followed by 30 sec at 98°C (denaturation), 45 sec at 55°C (annealing) and 1 min at 72°C (extension), and a final extension at 72°C for 10 min.

PCR products were purified using a PCR product purification kit (Shanghai Shengong, China). PCR products were electrophoretically size fractionated on 1.5% agarose gels that contained ethidium bromide and were visualized with UV light. The DNA fragment was gel-extracted using the DNA Gel Extraction Kit (Sangon Biotech). Purified PCR products were further used in the sequencing reaction process. The program used was an initial denaturation step at 96°C for 1 min, followed by 25 cycles (10 s of denaturation at 96°C, 50 s of annealing at 55°C, and 4 min of extension at 60°C) and a final extension at 60°C for 4 min. Cycle sequencing reactions were then purified using NaAc/EDTA precipitation. The sample was purified by ethanol and then dissolved in deionized formamide to undergo sequencing.

### Clinical and pathological data

The clinicopathological data of the patients with primary retroperitoneal EGIST were analyzed, including the pathological and medical (i.e., clinical features) and surgical records, pathological data (i.e., epithelioid or spindle), mitoses, and the presence of necrosis and hemorrhage. Mitoses were counted and summed from 50 high power fields (HPF). Gene mutations include those of exons 9, 10, 11, 13, 14, and 17 of the C-kit gene and those of exons 12 and 18 of PDGFRA gene. Adjuvant therapy, postoperative status, and prognosis (i.e., survival) were recorded during the follow-up, which was performed *via* telephone. The last follow-up date was February 28, 2022. The overall survival (OS) time was calculated from the date of operation to the date of death or the last follow-up date.

### Statistical analysis

Analyses were performed using SPSS Statistics version 25. Continuous variables are presented as the median (interquartile range) if the distributions were skewed or as the mean &amp;plusmn; standard deviation (SD).

## Results

### Baseline characteristics

Three patients were male and three female. The mean age was 64 (range: 48–77) years, the mean BMI was 21.9 (range: 17.9–25.1), and the average diameter of the primary tumor was 16.0 (range: 8.8–29.5) cm. One patient presented complications with moderate anemia, and one developed hypertension. The major symptoms were abdominal pain in two patients, no symptoms in two patients (abnormality was found during medical checkups), and a palpable mass in two patients.

### Treatment-related index

**Figure 1:**
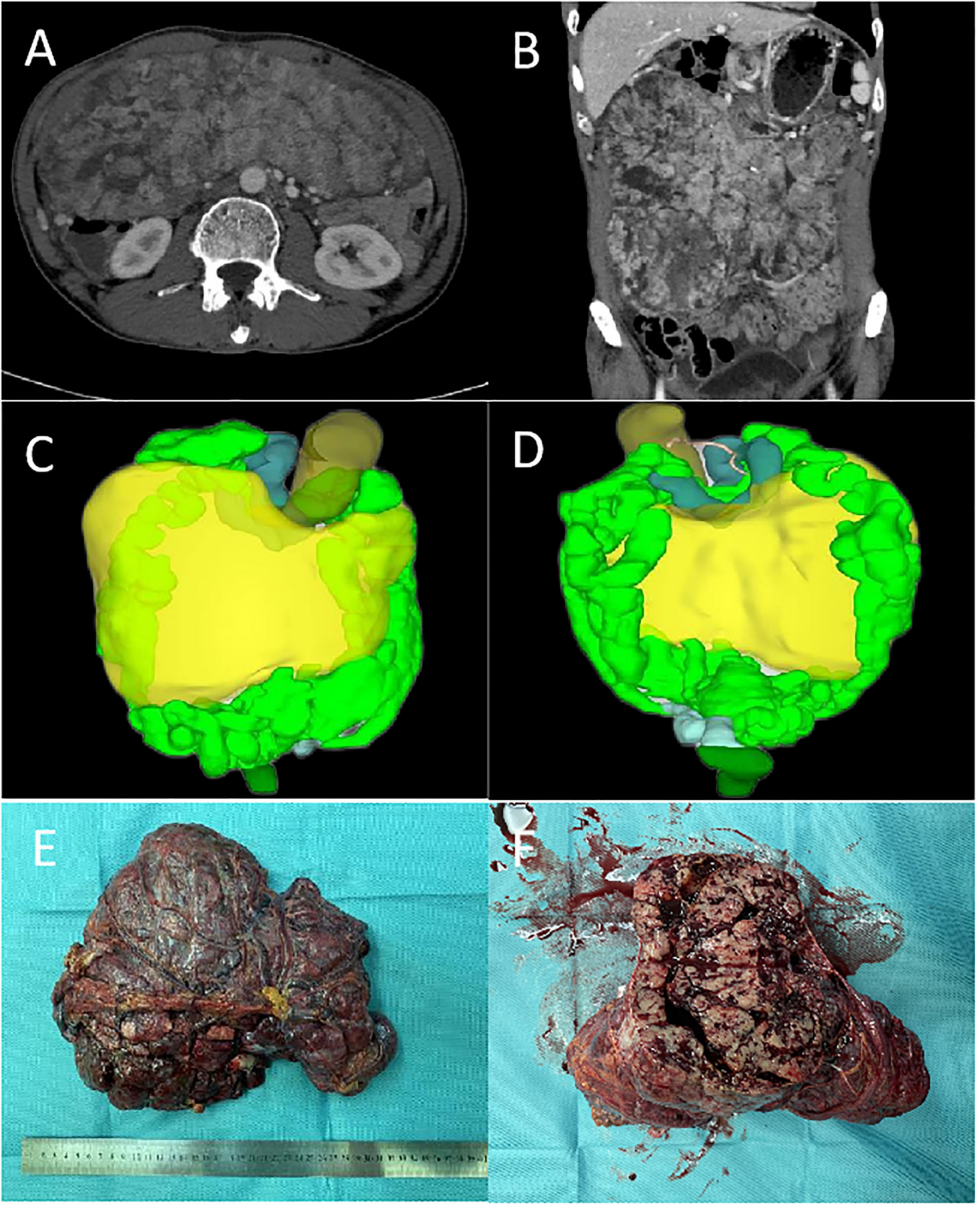
The representative pictures of tumors (Case 2): **(A, B)**, the cross-sectional and coronal views of CT. **(C, D)**, the front and back of the 3D visualization image. **(E, F)**, the resected tumor specimen.

In all patients, complete resection (R0/R1) was achieved. Representative pictures of the indicated tumors had been showed in **Figure 1** (Case 2). Five patients achieved en-block resection, and the other underwent anhydrous alcohol immersion in the abdominal cavity for 15s after complete resection due to tumor rupture. The median operation time was 170 (range: 130–261) min, and median intraoperative blood loss was 40 (range: 10–700) mL. The median hospital stay was 7.5 (range: 3-11) d and not postoperative complications occur in any of the patients. All patients received imatinib for three years as adjuvant therapy after the operation **(** **Table 1** **).**

**Table 1:** Baseline features of six patients with retroperitoneal EGISTs.

Case	1	2	3	4	5	6	Media
Gender	Male	Female	Male	Female	Male	Female	–
Year	64	77	61	55	67	72	65.50
BMI	21.5	24.4	25.0	20.8	21.8	17.9	21.65
Symptoms	Lump	No	No	No	Pain	Pain	–
Margin	R0/R1	R0/R1	R0/R1	R0/R1	R0/R1	R0/R1	R0/R1
Size (cm)	20	29.5	8.8	9.2	15	16.9	15.95
Modified NIH	High	High	High	High	High	High	–
Invasion	No	No	No	Liver	Mesocolon	Liver	–
Cell type	Spindle	Spindle	Spindle	Spindle	Spindle	Spindle	–
OS	109(a)	29 (a)	32 (a)	26 (a)	11 (d)	36(a)	30.5

a, alive; d, dead.

### CKI gene mutation and clinical pathological characteristics

**Figure 2:**
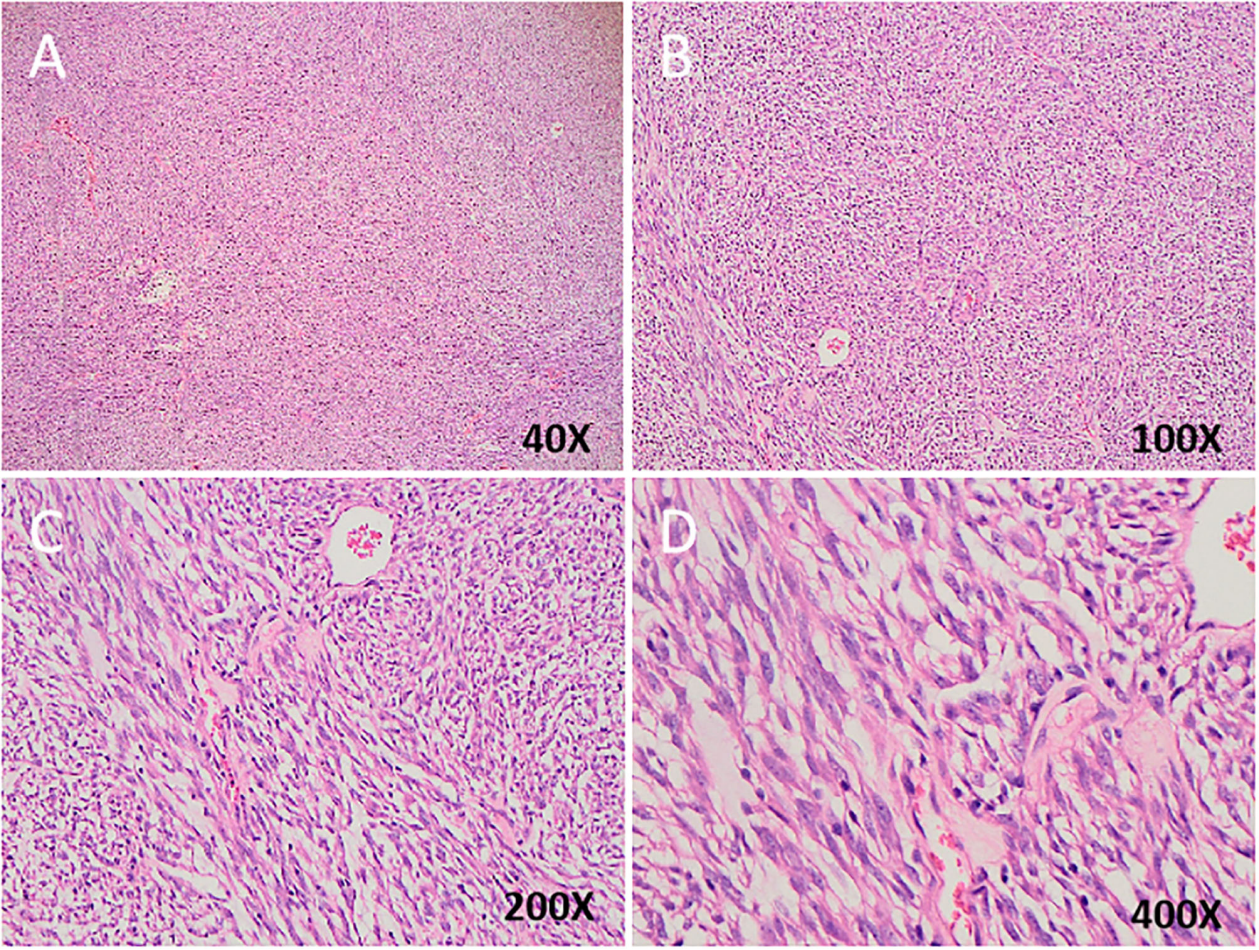
**(A–D)** Spindle tumor cells are arranged in fascicles with palisading pattern. Hematoxylin and eosin counterstain in different magnification.

Tumor tissues were also collected for HE staining after surgery, and the representative pathological change of the staining were displayed in **Figures 2A–D** (Case 4). Among these patients, there was rupture of tumor capsule in one case (patient 3), focal necrosis of tumor in four (patients 1, 2, 5, and 6), intratumoral hemorrhage in four (patients 2, 4, 5, and 6), and non-invasion of surrounding organs in three (patients 1, 2, and 3). The tumor was closely related to the adjacent intestinal wall in three cases (3, 4, and 5) and infiltrated to the liver in two cases (patients 4 and 6).

Pathological examination displayed no invasion of the intestinal wall in three cases (patients 3, 4, and 5). Two cases (patients 4 and 6) revealed focal involvement of the liver, and one (patient 5) had mesenteric vein invasion.

**Figure 3:**
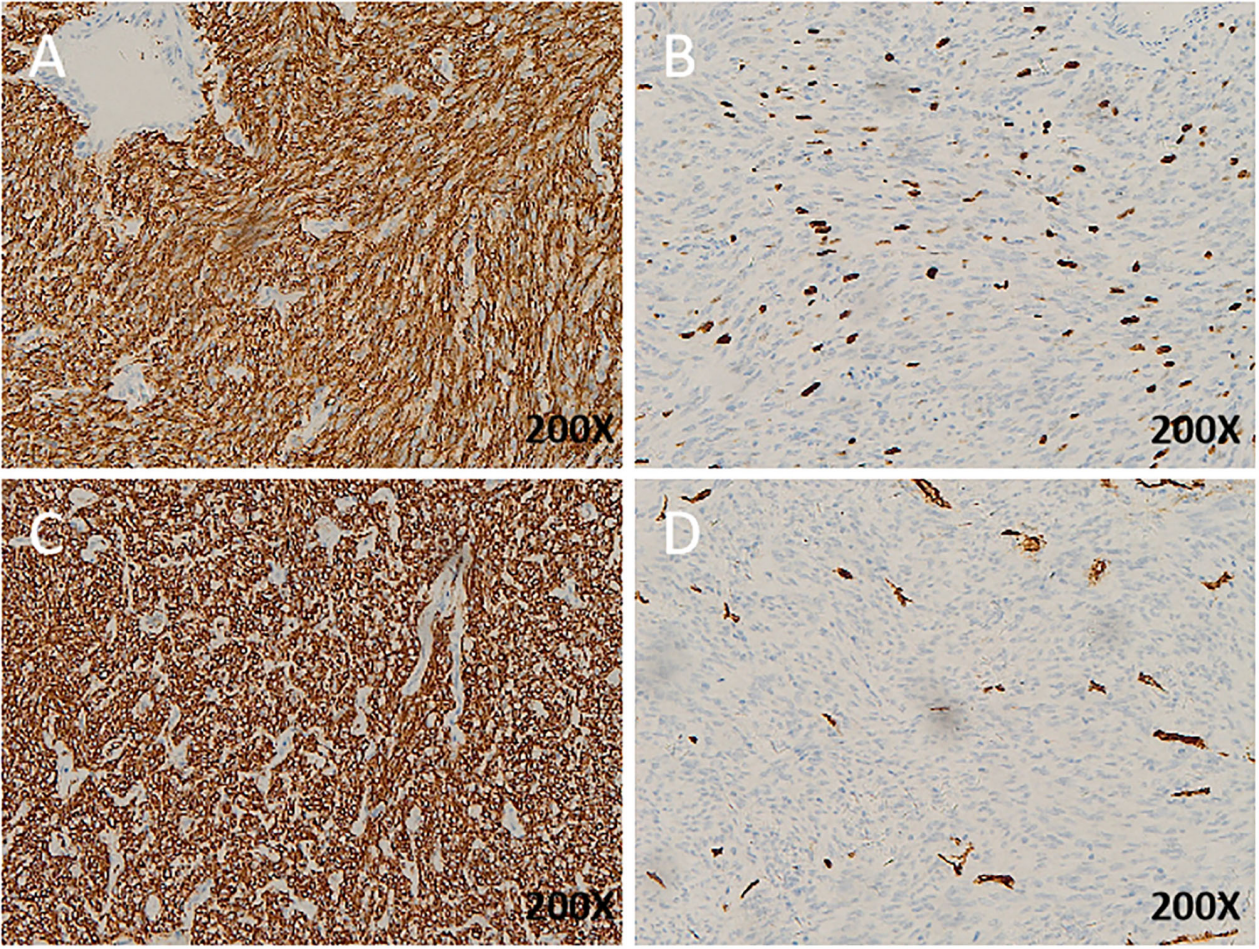
Immunohistochemistry (IHC) of EGIST: **(A)**, KI67 expression in EGIST; **(B)**, DOG1 expression in EGIST; **(C)**, cd34 expression in EGIST;**(D)**, CD117 expression in EGIST. All images with hematoxylin and eosin counterstain and ×200 magnification.

The immunohistochemical analysis demonstrated that desmin and S-100 were positive in all cases, while DOG1 and CD117 were negative in all cases. CD34 was positive in four cases (patients 1, 2, 4, and 5), while SMA was positive in four cases (patients 1, 3, 5, and 6). The mitotic index >5/50HPF was detected in four cases (patients 1, 4, 5, and 6) and Ki67 > 5% in three (patients 1, 4, and 5) **(** **Figure 3** and **Table 2** **)**.

**Table 2:** Pathological features of six patients with retroperitoneal EGISTs.

Case	1	2	3	4	5	6
Necrosis	+	+	–	–	+	+
Capsule	+	+	–	+	+	+
Hemorrhage	–	+	–	+	+	+
Cell type	Spindle	Spindle	Spindle	Spindle	Spindle	Spindle
DOG1	+	+	+	+	+	+
CD117	+	+	+	+	+	+
CD34	+	+	–	+	+	–
SMA	+	–	+	–	+	+
S-100	–	–	–	–	–	–
KI67	8%	5%	2%	10%	50%	1%
Mitoses (/50HPF)	>5	<5	<5	>5	>10	>5%

The symbol "+" means positive, "-" means negative.

**Figure 4:**
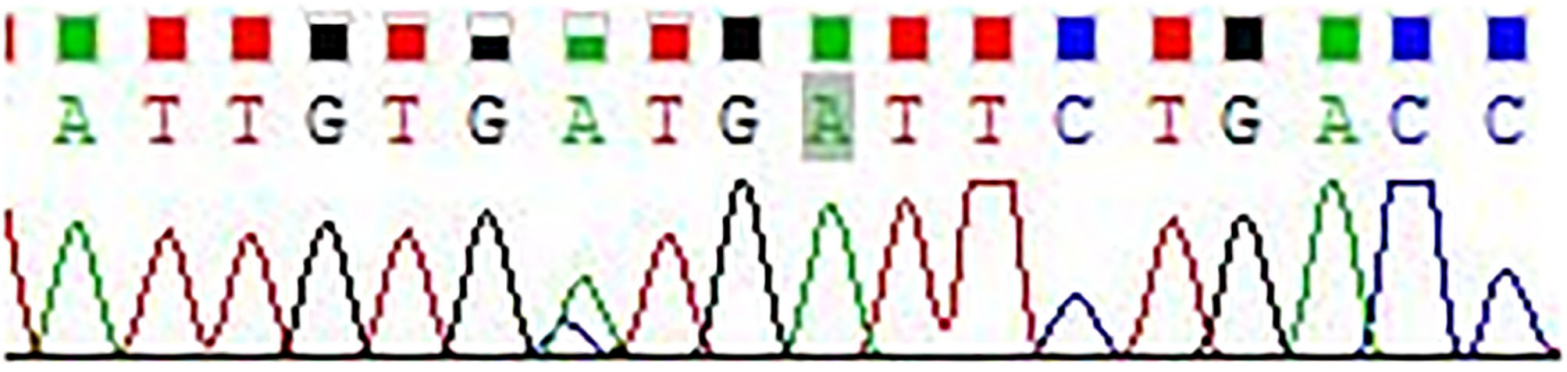
c-kit mutation at exon. Kit 10: 1621A>C (Met541Leu) missense mutation.

According to the modified National Institutes of Health (NIH) standard, the six cases of retroperitoneal EGIST were classified as high risk, and all were spindle cell tumors (10). CKI and PDGFRA genes were sequenced in all six patients, and eight loci (i.e., Exon9, Exon10, Exon11, Exon13, Exon14, Exon17, Exon12, and Exon18) were detected, including six cases of Exon11 mutation and two cases of Exon10 mutation (patients 4 and 5) **(** **Figure 4** and **Table 3** **)**.

**Table 3:** Kit g and PDGFRA gene mutation features of six patients with retroperitoneal EGISTs.

Case	1	2	3	4	5	6
Exon9	–	–	–	–	–	–
Exon10	–	–	–	+	+	–
Exon11	+	+	+	+	+	+
Exon13	–	–	–	–	–	–
Exon14	–	–	–	–	–	–
Exon17	–	–	–	–	–	–
Exon12	–	–	–	–	–	–
Exon18	–	–	–	–	–	–

The symbol "+" means positive, "-" means negative.

### Follow-up care

All cases were followed up for 6–24 months, and one patient died (patient 5) in 11 months after surgery.

## Discussion

GIST originates from the mesenchymal stem cell of the interstitial cells of Cajal, and it can expand to the whole gastrointestinal region. GIST was initially considered to originate in the gastrointestinal tract. However, tumors that resemble GIST have been found outside the gastrointestinal tract with similar immunohistological, pathological, and molecular features (1, 11). In 1999, a tumor report from the omentum and mesentery displayed similar histological and biological behavior to that of GIST. Since then, the term Extra-gastrointestinal stromal tumors (EGISTs) have been used (12). The origin of EGIST remains unclear. The proposed origins of EGIST are Cajal-like cells outside the intestinal wall or pluripotent stem cells outside the gastrointestinal tract; however, the parenteral tissue controversy is high (13–15). Most retroperitoneal EGISTs are large and The visceral peritoneum is weak. Therefore, retroperitoneal EGIST can Invade intraperitoneal organs, including the gastrointestinal tract (16, 17). Nonetheless, this does not necessarily mean that these tumors originate in the intestinal wall, considering that large retroperitoneal tumors such as liposarcoma often adhere to or invade the adjacent intestinal wall. In this study, only retroperitoneal EGISTs with postoperative pathology that did not invade the gastrointestinal tract were included. The immunophenotype and molecular characteristics of EGIST are also related to their tissue subtypes. Most spindle cells are CD117 positive and contain KIT mutations, while epithelioid cells with CD117 negative or PDGFRA mutants depict a more epithelial-like morphology (18, 19). In this study, all the tumors we evaluated were CD117 positive with KIT mutations and had a spindle-cell morphology, which supported this opinion.

Currently, C-kit gene detection is an efficient, sensitive, and reliable method for diagnosing retroperitoneal tumors. Although leiomyosarcoma may be c-Kit positive, there is no comparative c-Kit mutation in retroperitoneal soft tissue sarcoma. Therefore, we suggest c-Kit gene routine detection for difficult cases to distinguish from retroperitoneal sarcoma (8, 9, 18, 20). Recognizing the frequent presence of Kit gene or PDGFRA gene mutations in GIST will facilitate the accurate classification of retroperitoneal soft tissue tumors. Because the immunophenotype of EGIST is similar to its gastrointestinal counterpart, mutation detection can correct the diagnosis and determine the prognosis of EGIST. For KIT mutation sites, seven sites (i.e., Exon9, Exon11, Exon13, Exon14, Exon17, Exon12, and Exon18) are commonly detected. In GIST, the mutation probabilities of each c-Kit site are Exon11 (52%~58%), Exon18 (13% ~14%), Exon9 (6% ~ 9%), Exon12 (0.6% ~ 2%), Exon13 (1% ~ 3%), Exon14 (1% ~ 3%), and Exon17 (0% ~ 1%) (9). Zhang et al. (21) analyzed the relationship between c-Kit mutation and prognosis in 104 gastric stromal tumors and found that Exon11 mutation has better progression-free survival than Exon9 or wild type. In this study, the patients demonstrated Exon11 mutation, which may explain the good prognosis of the patients. Detailed information of these researches had been concluded in **Table 4** **(** 21–25 **)**. In addition to detecting international routine targets, we increased Exon10 testing and found mutations in two cases (patients 4 and 5), both of which invaded the surrounding tissue. Postoperative pathological analyses displayed that the tumor necrosis, mitotic index, and Ki67 were greater than the average levels. Patient 5 died due to tumor recurrence during the follow-up period. This seems to suggest that Exon10 or multi-site mutations may lead to a worse prognosis.

**Table 4:** Relationship between different mutation loci and prognosis.

Exons of mutation	mutation probabilities	Prognosis
Exon11	52%~58% (19)	Advanced GIST responds to imatinib at 34.5% and the PFS was 13–16.7 months. PFS at five years postoperatively without any adjuvant therapy was 41-63% (20, 21)
Exon18	13% ~14% (19)	PDGFRA exon 18 mutation is an indicator of a better prognosis. Avatinib has an objective remission rate (ORR) of 86% and PFS at five years postoperatively without any adjuvant therapy was 75%^22,23.^
Exon9	6% ~ 9% (19)	Advanced GIST responds to imatinib at 32.5% and the PFS was 24.7–39.4 months. PFS at five years postoperatively without any adjuvant therapy was 58% (20, 21).
Exon12	0.6% ~ 2% (19)	Due to its rarity, there is no literature describing its prognosis
Exon13	1% ~ 3% (19)	
Exon14	1% ~ 3% (19)	
Exon17	0% ~ 1% (19)	

Surgical resection is the primary standard treatment for non-metastatic EGIST. If a preoperative evaluation can completely remove the tumor, the curative treatment of EGIST is en bloc surgical resection with negative margins (15, 26). Surgical tumor removal is just part of the treatment, as postoperative chemotherapy, radiation therapy, targeted therapy, immune therapy, and follow-up are also important. However, there is no consensus on adjuvant therapy for EGIST. For patients who cannot be completely resected by preoperative evaluation, a biopsy should be performed, while preoperative imatinib treatment should be considered according to gene mutation status. For primary EGISTs, gene mutation detection is very important, which can provide a basis for the final diagnosis of the tumor and guide the molecular targeted therap. Therefore, low threshold suspicion is necessary significance (27). NCCN and ESMO guidelines recommend that genetic testing should be performed after surgery in high-risk cases of GIST (28, 29). If c-KIT and PDGFRa mutations are given adjuvant imatinib therapy for at least three years, this may also apply to retroperitoneal EGIST.

In this study, the tumor was completely removed in six patients and ruptured in one case (patient 3). For this ruptured tumor, we quickly washed the abdominal cavity with warm water at 50 °CC after soaking the tumor bed with anhydrous alcohol for 15 s. No recurrence or metastasis was observed 32 months after surgery; however, further follow-up was required. These may suggest that anhydrous alcohol can kill the residual tumor cells in the abdominal cavity, but when using anhydrous alcohol, the immersion time should be carefully monitored to avoid burning the normal tissue in the abdominal cavity. Combined organ resection was performed in four patients because the tumor had invaded the surrounding organs. In this study, mitosis >5/50HPF, Ki67 >5%, and tumor rupture was related to higher invasiveness, while tumor size did not correlate with invasiveness.

According to the modified National Institutes of Health (NIH) risk classification criteria for gastrointestinal stromal tumors, the six patients were pathologically identified as high-risk patients with Exon11 mutation. According to the ESMO guidelines, all patients were administered imatinib adjuvant therapy after the operation. All the patients were monitored, and only one patient died during the follow-up period. Compared with the literature, the prognosis of patients in this study is better, which may be as follows: 1. The length and diameter of the tumor are smaller than that reported. Gene detection of all patients revealed Exon11 mutation; 3. All patients were treated with imatinib after operation; 4. The sample size was small with no statistical significance (15, 21, 30, 31).

Our study had several limitations. Our study was a retrospective design, which means some biases cannot be avoided. Considering that the EGIST had a very low incidence and our sample size was small for each genotype, requiring a study with a larger sample size in the future to premature our conclusion.

In summary, retroperitoneal EGIST is very rare, highly malignant, and similar to GIST, making it difficult to differentiate from other mesenchymal tumors in the clinic. The tumor volume is often larger than that of GIST at the time of diagnosis. To diagnose this highly malignant tumor, GIST specialists may need to be aware of the possibility of EGIST. As no specific marker for the immunohistological diagnostic is available, when EGIST is not differentiated from other retroperitoneal tumor types, routine detection of the Kit or PDGFRA gene mutation should be performed to confirm the diagnosis and guide the follow-up treatment. Further, a mitosis >5/50HPF and Ki67 >5%, tumor rupture seems to be more aggressive, while tumor size has no relationship with invasiveness. Exon10 or multiple mutations may lead to a worse prognosis, and postoperative adjuvant therapy with imatinib mesylate may effectively prolong OS in patients. Given the rarity of retroperitoneal EGIST, more research is needed to fully understand its biological behavior and promote better treatment.

## Data availability statement

The original contributions presented in the study are included in the article/supplementary material. Further inquiries can be directed to the corresponding authors.

## Ethics statement

The studies involving human participants were reviewed and approved by Ethics Committee of Guangdong Provincial Hospital of Chinese Medicine. Written informed consent for participation was not required for this study in accordance with the national legislation and the institutional requirements.

## Author contributions

DD, XF, and JL designed the study; WLL, JW, and WJL contributed to the patient recruitment and collected the data. ZC and XT performed the statistical analysis. XL, HL, XY, and BZ contributed to administrative, technical, or material support. DD and JL wrote the manuscript. All authors contributed to the article and approved the submitted version.
